# Point-of-Care Ultrasound-Guided Management of Fulminant Influenza A(H3) with *Staphylococcus aureus* Necrotizing Pneumonia and Acute Cardiopulmonary Failure: A Case Report

**DOI:** 10.3390/healthcare14172766

**Published:** 2026-09-01

**Authors:** Luigi Vetrugno, Giovanni Serena, Stefania Buttera, Pierpaolo Accolla, Davide Pecori, Irene Batticci, Davide Stolfo, Massimo Imazio, Igor Vendramin, Flavio Bassi

**Affiliations:** 1 Anesthesia and Intensive Care Unit, Department of Emergency, Health Integrated Agency of Friuli Centrale, Tolmezzo Hospital, 33028 Tolmezzo, Italy; irene.batticci@asufc.sanita.fvg.it; 2 Department of Emergency, Academic Hospital of Udine, Health Integrated Agency of Friuli Centrale, Piazzale S. M. Della Misericordia, 15, 33100 Udine, Italy; giovanni.serena@asufc.sanita.fvg.it (G.S.); stefania.buttera@asufc.sanita.fvg.it (S.B.); pierpaolo.accolla@asufc.sanita.fvg.it (P.A.); flavio.bassi@asufc.sanita.fvg.it (F.B.); 3 Infectious Diseases Clinic, Department of Medicine (DMED), Azienda Sanitaria Universitaria Friuli Centrale (ASUFC), 33100 Udine, Italy; davide.pecori@asufc.sanita.fvg.it; 4 Cardiothoracic Department, Azienda Sanitaria Universitaria Friuli Centrale, University Hospital of Udine, 33100 Udine, Italy; davide.stolfo@asufc.sanita.fvg.it (D.S.); massimo.imazio@asufc.sanita.fvg.it (M.I.); igor.vendramin@asufc.sanita.fvg.it (I.V.)

**Keywords:** influenza A, *Staphylococcus aureus*, necrotizing pneumonia, septic cardiomyopathy, extracorporeal membrane oxygenation, Impella, point-of-care ultrasound

## Abstract

**Highlights:**

**What are the main findings?**

- Early suspicion of post-influenza *Staphylococcus aureus* superinfection is warranted in the presence of cytopenias, marked inflammation, haemoptysis, and rapidly worsening pulmonary infiltrates
- Early hub-and-spoke coordination and timely transfer to an ECMO-capable centre may be lifesaving in refractory respiratory or circulatory failure.

**What are the implications of the main findings?**

- Serial point-of-care ultrasound is useful for detecting evolving respiratory and circulatory failure and guiding escalation to mechanical support.
- Mechanical support should be individualized, including staged V-A ECMO, left ventricular unloading, and subsequent V-V ECMO when clinically indicated.

**Abstract:**

**Background:** Seasonal influenza is typically a self-limiting illness in healthy young adults, but severe and potentially life-threatening complications can occur. Influenza A infection may predispose patients to secondary bacterial infections, including *Staphylococcus aureus* pneumonia and bacteremia, which can rapidly progress to necrotizing pneumonia, acute respiratory distress syndrome (ARDS), and cardiovascular dysfunction. **Case Presentation:** We report the case of a young adult with influenza A(H3) infection complicated by *Staphylococcus aureus* bacteremia, necrotizing pneumonia, ARDS, and biventricular dysfunction. The patient experienced rapid cardiopulmonary deterioration requiring advanced critical care support, including extracorporeal membrane oxygenation (ECMO) and left ventricular unloading. Serial point-of-care ultrasound (POCUS) was central to clinical management, enabling early recognition of worsening respiratory and cardiac function and guiding timely escalation of supportive strategies. **Conclusions:** This case highlights the potential severity of influenza-associated complications even in young and previously healthy individuals. Repeated POCUS assessment can play a pivotal role in detecting rapid cardiopulmonary deterioration and guiding advanced interventions. Efficient hub-and-spoke organization may further support timely referral and management of critically ill patients requiring ECMO and specialized care.

## 1. Introduction

Seasonal influenza is commonly regarded as a benign, self-limiting disease in young adults without comorbidities [1]. Nonetheless, a minority of patients may develop severe complications such as ARDS, bacterial superinfection, septic shock, and myocardial dysfunction. Among bacterial pathogens, *Staphylococcus aureus* is particularly associated with necrotizing pneumonia and high mortality [2]. Influenza-related cardiac involvement may result from myocarditis or sepsis-induced cardiomyopathy. Clinical presentation may initially appear mild, with rapid deterioration occurring over a short time frame [3]. In this context, point-of-care ultrasound-guided management refers to the integrated use of bedside ultrasound techniques performed by treating clinicians—including lung ultrasound and both transthoracic and transesophageal echocardiography—to support real-time diagnostic assessment and therapeutic decision-making. This approach represents a valuable bedside tool for the early identification of evolving respiratory and circulatory failure [4,5,6,7]. Furthermore, timely referral to tertiary centers capable of providing advanced mechanical support, such as ECMO, is crucial. We describe a case of fulminant influenza A(H3) infection in a previously healthy young woman, emphasizing the role of serial ultrasound assessment and integrated critical care management.

## 2. Case Presentation

A 24-year-old woman with no significant medical history presented to a local emergency department after four days of influenza-like symptoms and a fever of up to 39 °C. She was initially discharged with symptomatic therapy but returned the following day because of worsening dyspnea, chest pain, nausea, and vomiting. On arrival, she was alert but markedly prostrated. Vital signs revealed sinus tachycardia (130 beats/min), blood pressure 115/50 mmHg, respiratory rate 20 breaths/min, oxygen saturation 99% on room air, and temperature 39.5 °C. Laboratory tests showed a WBC count of 8 × 10^3^/µL with lymphocytopenia (0.40 × 10^3^/µL), platelets 151 × 10^3^/µL, normal high-sensitivity troponin T, CRP 43 mg/L, and venous lactate 1.5 mmol/L. Electrocardiography demonstrated sinus tachycardia. Bedside lung ultrasound was unremarkable, and focused echocardiography showed preserved left ventricular systolic function with normal inferior vena cava collapsibility.

**Figure 1:**
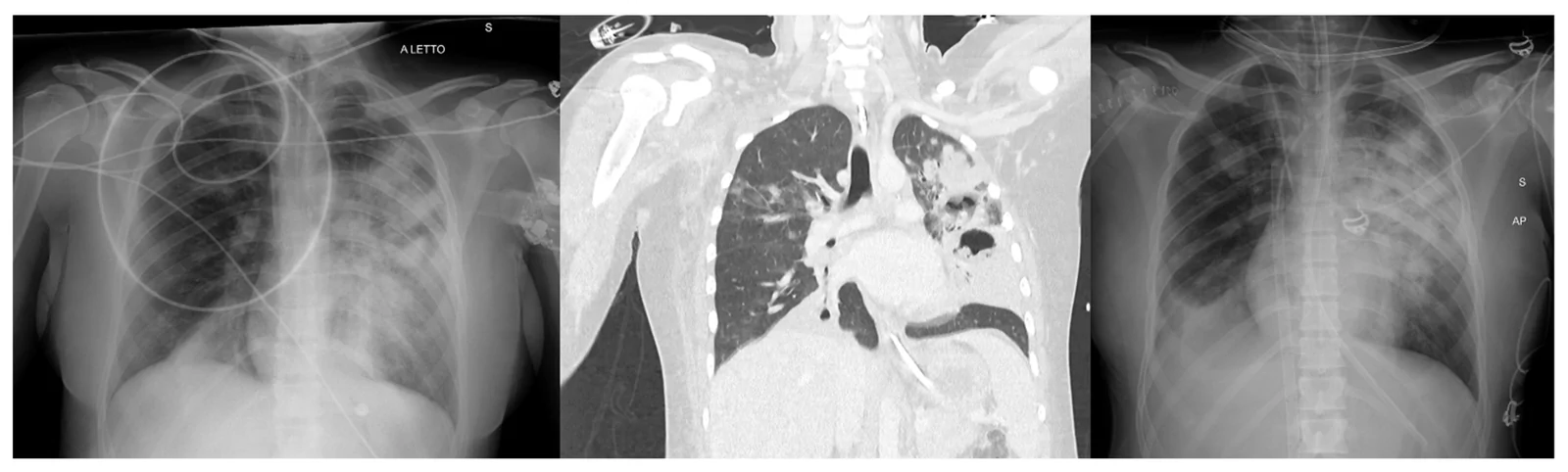
Radiological image of the case: (**left**) Initial chest radiograph demonstrating extensive left-sided pulmonary consolidation involving much of the left lung, consistent with pneumonia, without pleural effusion or pneumothorax; (**center**) coronal chest CT showing extensive left lung consolidation with cavitary change, consistent with abscess formation and overall in keeping with necrotizing pneumonia; (**right**) follow-up chest radiograph demonstrating an ECMO cannula projecting over the inferior vena cava in expected position; the remaining radiographic findings are otherwise unchanged.

Within hours, her condition deteriorated abruptly, with increasing dyspnea and hemoptysis. Repeat assessment showed tachycardia of 130 beats/min, respiratory rate 40 breaths/min, blood pressure 130/50 mmHg, and oxygen saturation 93%, requiring supplemental oxygen. Laboratory tests revealed leukopenia (3.2 × 10^3^/µL), persistent lymphopenia, CRP 130 mg/L, and procalcitonin 17 ng/mL. Radiological findings from the chest X-ray and CT scan are described and shown in Figure 1.

Empiric intravenous antibiotics (levofloxacin and ceftriaxone), hydrocortisone, and oseltamivir were initiated.

Despite therapy, respiratory failure rapidly progressed, prompting ICU admission and initiation of non-invasive ventilation. The patient developed lactic acidosis, progressive cytopenias, and hemodynamic instability requiring norepinephrine. Serial testing showed profound immune suppression, with severe lymphopenia, neutropenia, and thrombocytopenia [white cells 1730/µL (n.r. 4800–10,800/µL), total lymphocytes 420/µL (1500–400/µL), CD3^+^LympocytesT 324/µL (n.r. 1000–3200 µL, CD4^+^/CD3^+^ ratio 219/µL (500–2200/µL), NK CD56^+^CD16^+^/CD3^−^ 8 µL (n.r. 90–540/µL), Lymphocytes B CD19^+^ 88/µL (n.v. 105–560/µL)]. Following Infectious Diseases consultation, antimicrobial therapy was escalated to ceftaroline (600 mg intravenously every 12 h) and azithromycin (500 mg intravenously every 24 h), and IgM-enriched immunoglobulin was administered. Urgent transthoracic echocardiography revealed severe global left ventricular dysfunction (LVEF 15–20%) with a small pericardial effusion, raising suspicion for acute myocarditis or sepsis-induced cardiomyopathy (Supplemental Clip S1). Worsening respiratory and circulatory failure necessitated intubation and transfer to a tertiary referral center.

**Figure 2:**
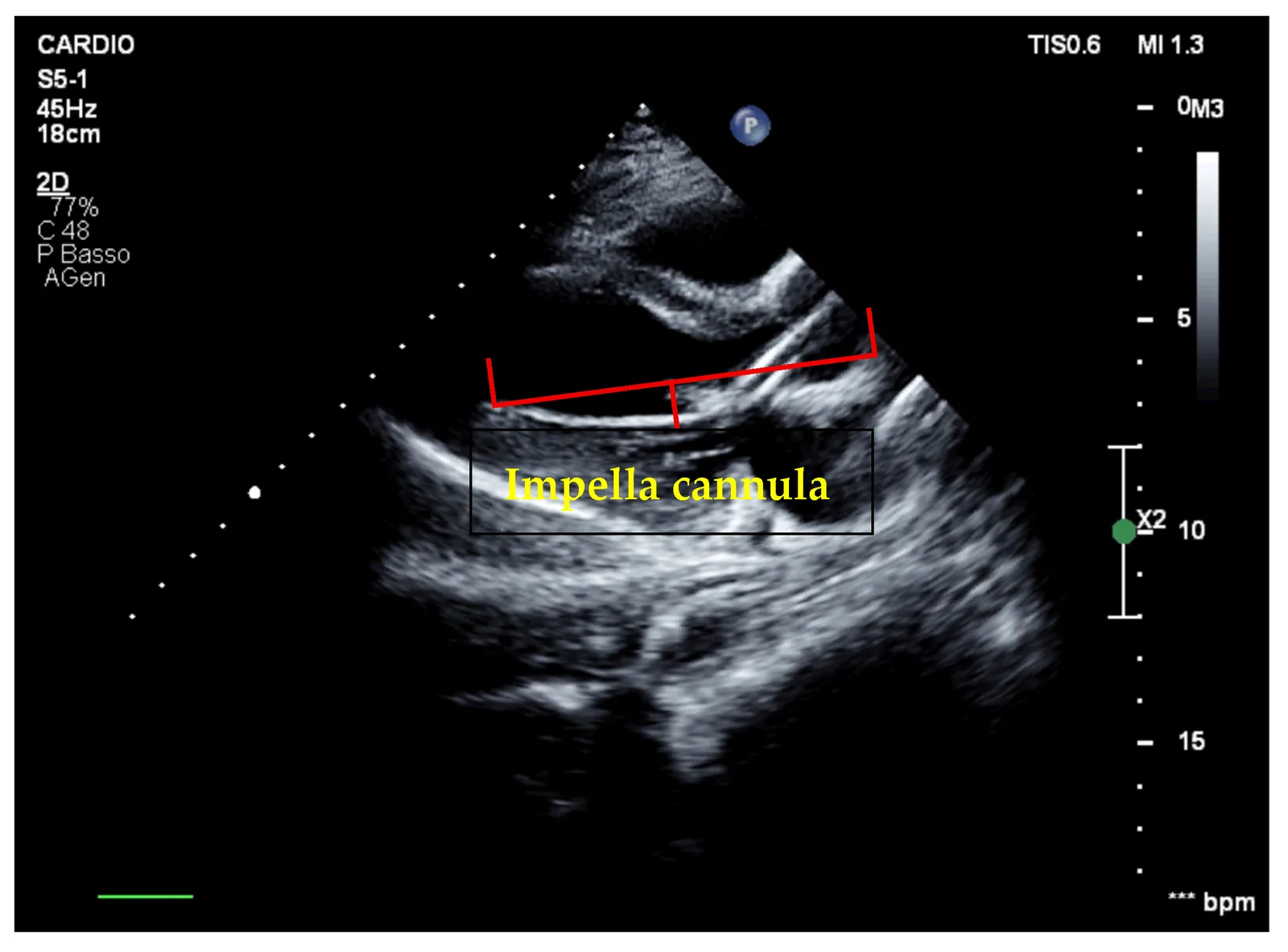
Echocardiography parasternal long-axis view showing the correct positioning of the IMPELLA system.

On arrival, the patient exhibited severe cardiorespiratory instability. Arterial blood gas analysis showed respiratory acidosis and profound hypoxemia (PaO_2_/FiO_2_ ratio 56), consistent with severe ARDS (CT scan Figure 1—centre panel). Bronchoscopy revealed erythematous, friable bronchial mucosa with abundant secretions. Inhaled nitric oxide produced minimal improvement. Transthoracic echocardiography confirmed biventricular dysfunction, and intravenous adrenaline and vasopressin were added to the ongoing norepinephrine infusion. Cardiac and chest ultrasound were performed using Philips Affiniti 70 ultrasound system (Philips Healthcare, Best, The Netherlands) equipped with a C5-1 convex transducer (1–5 MHz), S5-1 phased-array probe (1–5 MHz) or X7-2t transoesophageal probe (2–7 Mhz). Daily point-of-care cardiac (transthoracic or transoesophageal) ultrasound was performed by intensive care physician. Point-of-care lung ultrasound was performed by intensive care physician when clinically indicated (Supplemental Figure S1). Because no meaningful improvement was achieved despite maximal ventilatory and hemodynamic support, escalation to mechanical circulatory support was undertaken, and femoro-axillary veno-arterial extracorporeal membrane oxygenation (V-A ECMO) was implanted (Figure 1—right panel). On the following day, persistent severe ventricular dysfunction was observed, with left ventricular dilation, akinetic apex, rhythm instability due to atrial fibrillation, and an estimated LVEF of 5%, together with increasing arterial pressure despite discontinuation of vasoactive drugs. After coronary angiography excluded significant coronary artery disease, a microaxial flow pump (Impella) was implanted for left ventricular unloading (Figure 2). Concomitant endomyocardial biopsy demonstrated severe, diffuse edema associated with scattered foci of mild lymphomonocytic inflammatory infiltrate, with occasional eosinophils; rare microfoci of myocyte degeneration; and absence of overt necrosis. The morphological findings are suggestive of mild-to-borderline lymphocytic myocarditis, later interpreted in the context of septic shock. Blood cultures grew *Staphylococcus aureus*, and daptomycin was added; bronchoalveolar lavage confirmed influenza A(H3).

Levosimendan was administered, with gradual cardiac recovery (Supplemental Clip S2).

On day 3, V-A ECMO was converted to veno-venous ECMO (V-V ECMO), reflecting hemodynamic recovery with persistent respiratory failure. The Impella device was removed on day 5, and ECMO support was definitively discontinued on day 8 without complications. Follow-up imaging revealed extensive pulmonary consolidation with cavitary lesions consistent with necrotizing pneumonia; antibiotic therapy was switched to linezolid (Figure 1 centre and right panel). Transesophageal echocardiography and funduscopic examination showed no evidence of metastatic *S. aureus* dissemination.

A formal transthoracic echocardiographic examination was performed on Day 12, after recovery of ventricular function following discontinuation of ECMO and Impella support. The cardiologist reported a left ventricle of normal size and wall thickness, with preserved regional wall motion and normal systolic function, with a left ventricular ejection fraction of 65%. The diastolic filling pattern and filling pressures were within normal limits. The right ventricle was of normal size, with preserved wall motion and systolic function. Biatrial dimensions were normal. Mild mitral regurgitation, mild-to-moderate tricuspid regurgitation, and pulmonary regurgitation were noted. Estimated pulmonary artery pressure was at the upper limit of normal, at 48 mmHg. The inferior vena cava was of normal size and showed normal inspiratory collapsibility. No pericardial effusion was detected. No echocardiographic findings suggestive of infective endocarditis vegetations were observed.

**Figure 3:**
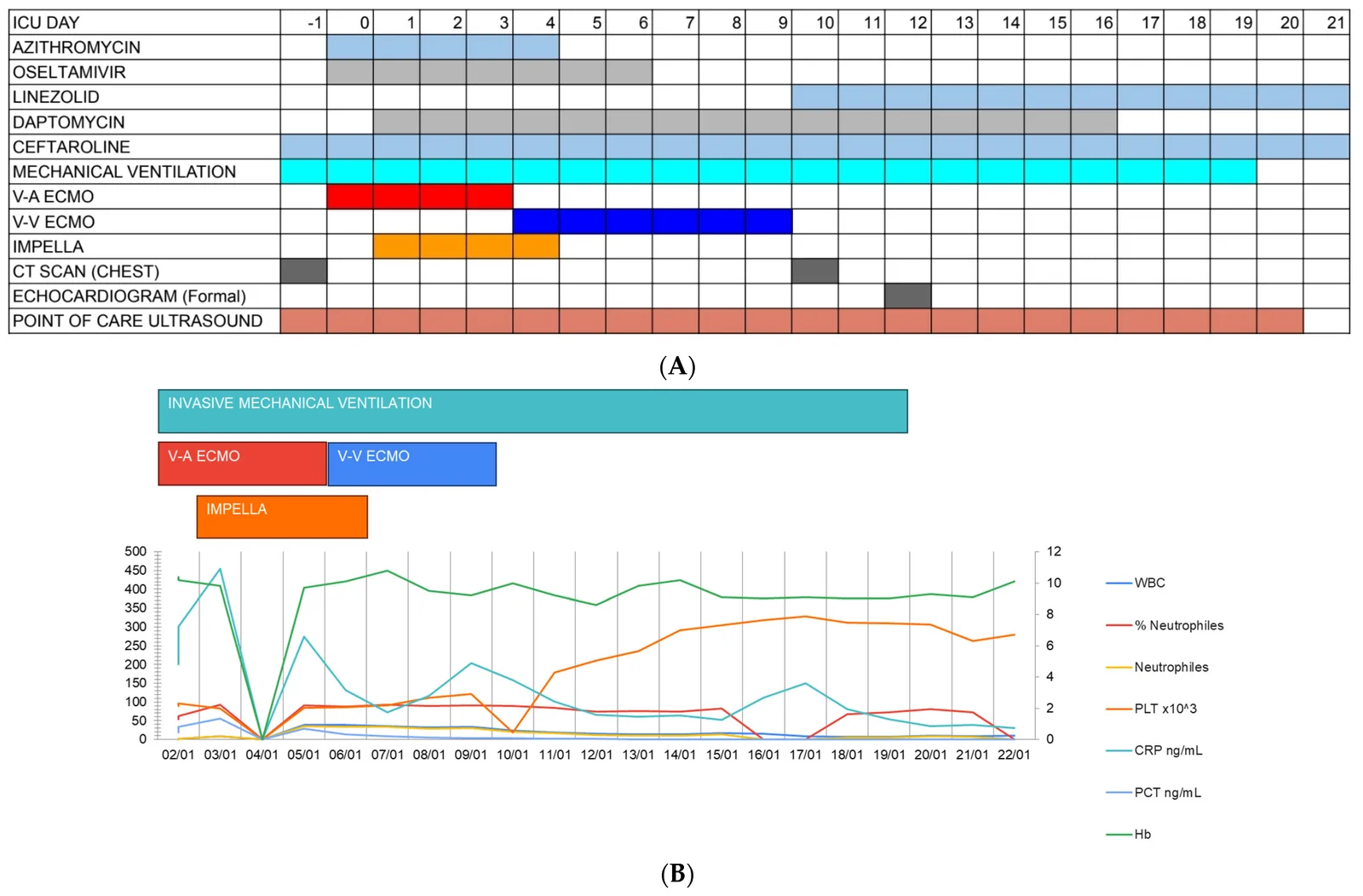
The Gantt chart (**A**) and a visual picture (**B**) illustrates day-by-day changes in patient management.

The patient remained mechanically ventilated until day 18, when she was successfully extubated after two previous failed attempts related to ineffective cough. Thereafter, she was managed with high-flow oxygen therapy, maintaining adequate gas exchange and satisfactory respiratory mechanics, with easily mobilized secretions. Figure 3A,B show the Gantt chart and the duration of mechanical support for the patient in the ICU over time.

A new fever episode occurred on day 29. Bronchoalveolar lavage (BAL) cultures were positive for *Pseudomonas aeruginosa*; however, given the declining C-reactive protein and procalcitonin levels, this was considered colonization, and no new antibiotic therapy was initiated.

After further clinical improvement, she was transferred to the pulmonology ward and was subsequently discharged home in good clinical condition. Cardiac MRI, performed in the post- hospitalization setting on day 51, showed intramyocardial late gadolinium enhancement involving the mid-septal segments and anterior wall, consistent with sequelae of acute myocarditis, with preserved biventricular systolic function.

Although these findings may have been compatible with myocarditis, they were not considered sufficiently specific to establish a definitive diagnosis.

## 3. Discussion

This case illustrates the potentially fulminant course of *Influenza A* infection in a previously healthy young adult and highlights the combined impact of viral pneumonia, bacterial superinfection, severe ARDS, and acute cardiac dysfunction. It also underscores the importance of coordinated management between a peripheral spoke hospital and a tertiary referral center, where advanced respiratory and circulatory support can be delivered in a timely fashion. A first notable aspect of this case is the deceptively mild initial presentation. At first reassessment, despite fever, chest symptoms, and marked asthenia, the patient maintained normal oxygen saturation on room air, had no signs of overt shock, and showed preserved ventricular systolic function on bedside echocardiography. Yet within hours she developed hemoptysis, hypoxemia, diffuse pulmonary involvement, and rapidly progressive systemic inflammation. This abrupt progression has been described in severe influenza, where early clinical findings may underestimate the subsequent severity of the host inflammatory response [1,2]. In our patient, the rapid development of leukopenia and lymphopenia is especially relevant. These abnormalities have been associated with severe influenza and poorer outcomes, likely reflecting impaired antiviral defense and profound immune dysregulation. The severe lymphocyte depletion later confirmed by immunophenotyping further supports the presence of marked host immune dysfunction. The coexistence of *Influenza* A(H3) infection and *Staphylococcus aureus* bacteremia strongly supports the diagnosis of post-influenza bacterial superinfection. This relationship is well established in the literature. Influenza virus damages the respiratory epithelium, impairs mucociliary clearance, and alters innate immune function, especially macrophage- and neutrophil-mediated antibacterial responses, thereby facilitating secondary bacterial invasion [1,2]. The isolate was confirmed as MSSA, with low MICs for daptomycin and linezolid. Empirical combination therapy was initially chosen to maximize bactericidal activity and promote rapid bloodstream clearance. Daptomycin was used for bacteraemia as it does not achieve adequate pulmonary penetration, whereas linezolid was selected for staphylococcal pneumonia owing to its excellent epithelial lining fluid penetration [3]. Among the bacterial pathogens associated with severe post-influenza pneumonia, *S. aureus* is particularly aggressive and has been repeatedly linked to rapidly progressive community-acquired pneumonia, necrotizing lung injury, cavitation, hemoptysis, septic shock, and high mortality in otherwise healthy patients [6,7].

The evolution in our patients, including cavitary pulmonary lesions on follow-up CT and the absence of a surgical indication, was fully consistent with this pattern.

Another key feature of this case is the development of profound cardiovascular dysfunction. Initial bedside echocardiography at the local hospital was normal, but serial reassessment documented severe left ventricular impairment, subsequently progressing to biventricular dysfunction. This dynamic evolution emphasizes the importance of repeated ultrasound evaluation in unstable patients with influenza-related respiratory failure. Influenza-associated myocardial involvement, including myocarditis, stress-related cardiomyopathy, and sepsis-induced myocardial depression, is uncommon but well recognized [7,8,9]. In this patient, the endomyocardial biopsy showed mild-to-borderline lymphocytic myocarditis, while the overall clinical course and microbiological findings supported a major contribution of septic cardiomyopathy. Rather than representing mutually exclusive diagnoses, these mechanisms may have coexisted and amplified each other, resulting in severe but reversible myocardial dysfunction. The use of point-of-care ultrasound and echocardiography was central throughout the clinical course. At the spoke hospital, focused echocardiography first helped exclude overt cardiac dysfunction, then later identified severe ventricular impairment and supported transfer to the tertiary center. At the referral center, echocardiography guided the decision to initiate V-A ECMO by documenting a degree of circulatory failure that could not be managed by isolated respiratory support alone. Subsequent imaging was equally important in identifying left ventricular dilation and inadequate unloading, thereby prompting implantation of an Impella device. In that way the decision to initiate Impella support was based on evidence of left ventricular overload and inadequate unloading, including left ventricular distension, absent or infrequent aortic valve opening, pulmonary edema, reduced pulse pressure, and intracardiac blood stasis. Similarly, conversion from VA-ECMO to VV-ECMO was considered after recovery of ventricular function and stabilization of the patient’s cardiac and hemodynamic status, as demonstrated by adequate cardiac output, improved arterial pulsatility, regular aortic valve opening, and no ongoing need for mechanical circulatory support.

Ebert and colleagues reported a similar case involving a young woman who experienced rapid clinical deterioration following influenza A infection. She required VV–VVA ECMO support, followed by combined ECMO and Impella therapy (ECMELLA), with device explantation occurring within a timeframe comparable to that observed in our patient [10].

Nandate et al. described two cases of fulminant myocarditis complicated by refractory cardiogenic shock that were successfully managed using combined VA-ECMO and Impella support [11]. This strategy represents a promising approach for patients with fulminant myocarditis and cardiogenic shock because it combines the systemic hemodynamic support provided by VA-ECMO with direct left ventricular unloading provided by the Impella device.

By improving left ventricular decompression and reducing ventricular wall stress, this combined approach may also reduce myocardial oxygen demand and limit the need for inotropic and vasoactive medications during the acute phase of myocardial injury.

Consistent with the findings in our case, the authors reported favorable clinical outcomes, including substantial recovery of left ventricular function within one week.

Serial echocardiographic assessment then informed the transition from V-A ECMO to V-V ECMO as cardiac function improved and finally supported safe removal of mechanical support. This sequence illustrates how ultrasound is not merely a diagnostic adjunct, but rather a continuous bedside decision-making tool in complex critical care pathways. From an organizational perspective, the case also demonstrates the value of an effective hub-and-spoke network. Once rapid deterioration was recognized, timely communication between the peripheral hospital and the tertiary center allowed escalation of care before irreversible multiorgan failure developed. This is consistent with the previous literature showing that patients with severe influenza-associated ARDS benefit from early referral to centers experienced in ECMO and advanced critical care [12,13]. The positive outcome in our patient was likely related not only to the availability of extracorporeal support itself, but also to the timing of referral, the integration of microbiological, cardiological, and intensive care expertise, and the capacity to tailor support dynamically as the dominant pathophysiological process shifted from combined cardiopulmonary failure to isolated respiratory dysfunction. The pattern observed in this case is in keeping with similar reports in the literature. Severe influenza complicated by *S. aureus* coinfection has repeatedly been associated with necrotizing pneumonia and rapid progression to ARDS, particularly in young and previously healthy patients [2,3]. Likewise, influenza-associated myocarditis and acute reversible ventricular dysfunction have been reported in young adults and children, sometimes requiring temporary mechanical circulatory support [8,9]. Finally, multicenter observational studies from the Influenza A(H1N1) pandemic demonstrated that extracorporeal support could achieve favorable outcomes in selected patients with refractory influenza-related respiratory failure when delivered at experienced centers [13,14,15,16,17].

Taken together, this case reinforces several practical clinical messages. First, severe influenza should remain in the differential diagnosis of rapidly progressive respiratory failure even in young, previously healthy adults, and apparent early stability should not provide false reassurance. Second, the combination of lymphopenia or leukopenia, high inflammatory markers, hemoptysis, and rapidly progressive pulmonary infiltrates should prompt early suspicion of bacterial superinfection, particularly *S. aureus*. Third, serial bedside ultrasound is invaluable for identifying evolving cardiac involvement and guiding the selection and adjustment of advanced support strategies. Finally, efficient spoke-to-hub communication and early transfer to a tertiary center may be lifesaving in patients with combined refractory respiratory and circulatory failure.

## 4. Conclusions

This case of influenza A(H3) complicated by *Staphylococcus aureus* bacteremia, necrotizing pneumonia, severe ARDS, and acute myocardial dysfunction highlights how rapidly a common viral illness can evolve into a multisystem critical illness. Early recognition of deterioration, repeated ultrasound assessment, prompt antimicrobial escalation, and timely transfer within a hub-and-spoke network enabled successful use of V-A ECMO, left ventricular unloading, and subsequent V-V ECMO. The case supports the role of integrated multidisciplinary management in improving outcomes in fulminant influenza-associated cardiorespiratory failure.

## Data Availability

A minimum dataset has been included as a single structured table in the Supplementary Material.

## Supplementary Materials

The following supporting information can be downloaded at https://www.mdpi.com/article/10.3390/healthcare14172766/s1, Figures S1 and S2; Clip S1: Baseline echocardiography at ICU admission (transthoracic echocardiography performed at ICU admission, demonstrating biventricular dilatation with severely impaired systolic function), Clip S2: Transthoracic echocardiography performed after transfer to the hub hospital, showing improved ventricular systolic function and Clip S3: Recovery after mechanical circulatory support weaning (transthoracic echocardiography performed after weaning from mechanical circulatory support, showing complete recovery of left ventricular systolic function).

## Abbreviations

**Abbreviation**	**Meaning**
**A(H3)**	Influenza A subtype H3
**ARDS**	Acute respiratory distress syndrome
**CD**	Cluster of differentiation
**CRP**	C-reactive protein
**CT**	Computed tomography
**ECG**	Electrocardiography/electrocardiogram
**ECMO**	Extracorporeal membrane oxygenation
**ICU**	Intensive care unit
**IgM**	Immunoglobulin M
**IV**	Intravenous
**LVEF**	Left ventricular ejection fraction
**NK**	Natural killer
**POCUS**	Point-of-care ultrasound
**SARS-CoV-2**	Severe acute respiratory syndrome coronavirus 2
**V-A ECMO**	Venoarterial extracorporeal membrane oxygenation
**V-V ECMO**	Venovenous extracorporeal membrane oxygenation
**WBC**	White blood cell(s)
**n.r.**	Normal range
**n.v.**	Normal value
**T**	Temperature
**µL**	Microliter
**mg/L**	Milligrams per liter
**ng/mL**	Nanograms per milliliter
**mmHg**	Millimeters of mercury
**bpm**	Beats per minute
**CD3+**	Cluster of differentiation 3–positive T lymphocytes
**CD4+/CD3+**	CD4-positive/CD3-positive T-cell ratio
**CD19+**	Cluster of differentiation 19–positive B lymphocytes
**CD56+CD16+/CD3−**	Natural killer-cell phenotype
**CXR**	Chest X-ray/radiograph
**IVC**	Inferior vena cava
